# Supplementary material for: Role and mechanism of NCAPD3 in promoting malignant behaviors in gastric cancer
Source: Front Pharmacol. 2024 Apr 22;15:1341039. doi: 10.3389/fphar.2024.1341039 (PMC11070777; doi:10.3389/fphar.2024.1341039)
Supplement: Supplementary file 11 [file DataSheet2.ZIP › GSEA/Transcipt factor/my_analysis.Gsea.1599461814734/FOXO4_01.html]

Details for gene set FOXO4\_01[GSEA]

|  || Dataset | filtered\_dataset.sample\_info.cls#WT\_versus\_NCAPD3\_MUT |
| Phenotype | sample\_info.cls#WT\_versus\_NCAPD3\_MUT |
| Upregulated in class | NCAPD3\_MUT |
| GeneSet | FOXO4\_01 |
| Enrichment Score (ES) | -0.5176175 |
| Normalized Enrichment Score (NES) | -2.4322934 |
| Nominal p-value | 0.0 |
| FDR q-value | 0.007964022 |
| FWER p-Value | 0.024 |
Table: GSEA Results Summary

  

Fig 1: Enrichment plot: FOXO4\_01      
 Profile of the Running ES Score & Positions of GeneSet Members on the Rank Ordered List

  

| SYMBOL | TITLE | RANK IN GENE LIST | RANK METRIC SCORE | RUNNING ES | CORE ENRICHMENT || 1 | 51134 | CEP83 | 19 | 1.038 | 0.0669 | No |
| 2 | 805 | CALM2 | 423 | 0.518 | -0.1829 | No |
| 3 | 8904 | CPNE1 | 814 | 0.308 | -0.4396 | No |
| 4 | 667 | DST | 886 | -0.303 | -0.4672 | No |
| 5 | 9414 | TJP2 | 916 | -0.345 | -0.4612 | No |
| 6 | 7067 | THRA | 976 | -0.392 | -0.4733 | No |
| 7 | 638 | BIK | 1024 | -0.421 | -0.4744 | No |
| 8 | 23764 | MAFF | 1039 | -0.436 | -0.4507 | No |
| 9 | 54541 | DDIT4 | 1133 | -0.502 | -0.4787 | Yes |
| 10 | 639 | PRDM1 | 1159 | -0.523 | -0.4561 | Yes |
| 11 | 27250 | PDCD4 | 1170 | -0.537 | -0.4216 | Yes |
| 12 | 2119 | ETV5 | 1176 | -0.545 | -0.3829 | Yes |
| 13 | 153222 | CREBRF | 1187 | -0.559 | -0.3467 | Yes |
| 14 | 861 | RUNX1 | 1293 | -0.687 | -0.3690 | Yes |
| 15 | 55691 | FRMD4A | 1295 | -0.689 | -0.3163 | Yes |
| 16 | 3892 | KRT86 | 1313 | -0.724 | -0.2723 | Yes |
| 17 | 3084 | NRG1 | 1320 | -0.739 | -0.2194 | Yes |
| 18 | 57561 | ARRDC3 | 1324 | -0.744 | -0.1638 | Yes |
| 19 | 9990 | SLC12A6 | 1379 | -0.922 | -0.1311 | Yes |
| 20 | 10397 | NDRG1 | 1380 | -0.925 | -0.0594 | Yes |
| 21 | 55729 | ATF7IP | 1392 | -1.034 | 0.0129 | Yes |
Table: GSEA details [plain text format]

  

Fig 2: FOXO4\_01      
 Blue-Pink O' Gram in the Space of the Analyzed GeneSet

  

Fig 3: FOXO4\_01: Random ES distribution      
 Gene set null distribution of ES for **FOXO4\_01**
